# Gene Therapy Applications of Non-Human Lentiviral Vectors

**DOI:** 10.3390/v12101106

**Published:** 2020-09-29

**Authors:** Altar M. Munis

**Affiliations:** Nuffield Division of Clinical Laboratory Sciences, Radcliffe Department of Medicine, University of Oxford, Oxford OX3 9DU, UK; altar.munis@ndcls.ox.ac.uk; Tel.: +441865221844

**Keywords:** gene therapy, lentivirus, lentiviral vector, HIV-1, SIV, FIV, EIAV, non-primate lentivirus

## Abstract

Recent commercialization of lentiviral vector (LV)-based cell therapies and successful reports of clinical studies have demonstrated the untapped potential of LVs to treat diseases and benefit patients. LVs hold notable and inherent advantages over other gene transfer agents based on their ability to transduce non-dividing cells, permanently transform target cell genome, and allow stable, long-term transgene expression. LV systems based on non-human lentiviruses are attractive alternatives to conventional HIV-1-based LVs due to their lack of pathogenicity in humans. This article reviews non-human lentiviruses and highlights their unique characteristics regarding virology and molecular biology. The LV systems developed based on these lentiviruses, as well as their successes and shortcomings, are also discussed. As the field of gene therapy is advancing rapidly, the use of LVs uncovers further challenges and possibilities. Advances in virology and an improved understanding of lentiviral biology will aid in the creation of recombinant viral vector variants suitable for translational applications from a variety of lentiviruses.

## 1. Introduction to Lentiviral Vectors

The field of gene and cell therapy is advancing rapidly with lentiviral-based vectors being the preferred vector of choice due to their ability to infect both dividing and non-dividing cells and integrate transgenes into the host cell genome [1]. Lentiviruses belong to the Orthoretroviridae subfamily of the genus retroviruses and are characterized by their long latent incubation periods with low levels of viral pathogenicity [2].

They are divided into two major classes: primate and non-primate lentiviruses [3]. The primate lentiviruses include human immunodeficiency virus type 1 (HIV-1) and 2 (HIV-2) and simian immunodeficiency virus (SIV). The HIV-1 pandemic in the twentieth century and the resulting acquired immune deficiency syndrome (AIDS) instigated extensive research into the virus and uncovered crucial information regarding viral genome organization, replication, and life cycle, paving the way for the generation of HIV-1-based lentiviral vectors (LVs). The prototype virus for the non-primate lentiviruses is the ovine visna-maedi virus (VMV) [4]. Other members include feline immunodeficiency virus (FIV) [5,6], equine infectious anemia virus (EIAV) [7], caprine arthritis encephalitis virus (CAEV) [8], bovine immunodeficiency virus (BIV) [9], and jembrana disease virus (JDV) [10].

### 1.1. Retroviral Biology

Retroviruses are enveloped RNA viruses containing two copies of a positive-sense single-stranded RNA genome [11]. The hallmark of this family of viruses is the reverse transcription and integration steps [12]. Reverse transcriptase encoded by viral genome transcribes viral RNA to synthesize double-stranded DNA, which is then integrated into the host cell genome by the viral integrase (defined as provirus) [13].

**Figure 1:**
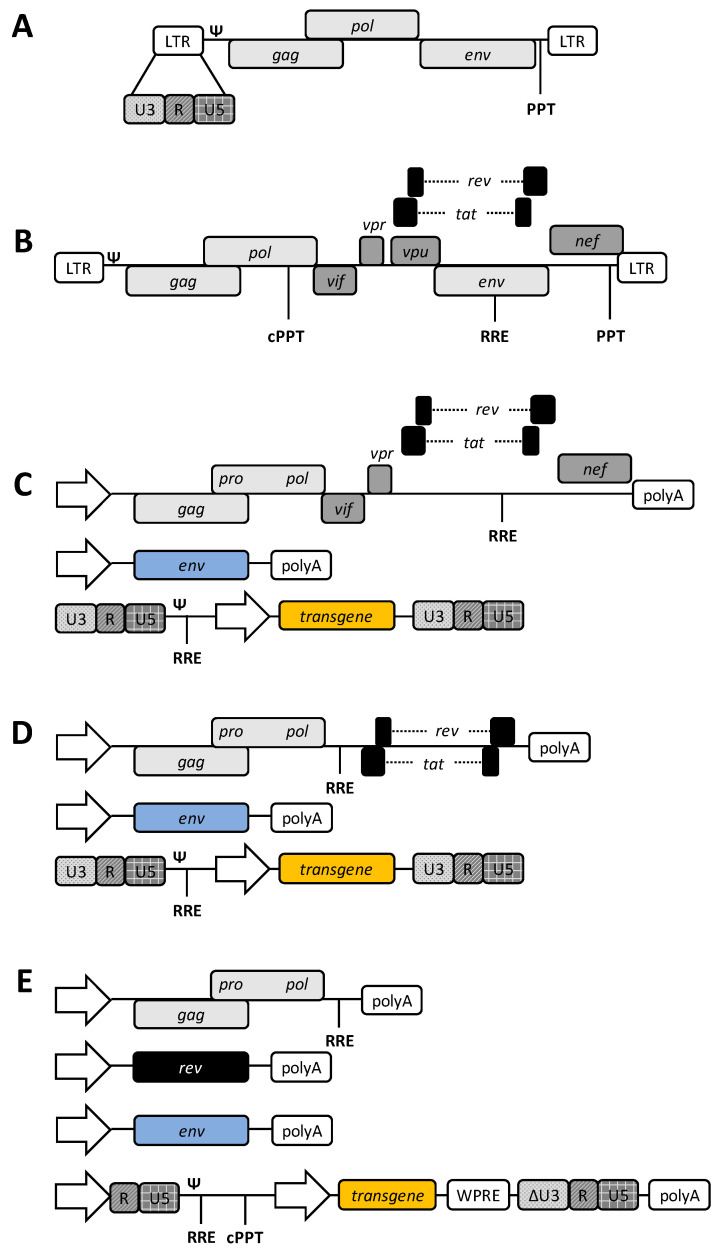
Engineering of HIV-1-based lentiviral vector (LV) systems. Wild-type (wt) genomes of (**A**) Moloney murine leukemia virus, a simple retrovirus, and (**B**) HIV-1. Essential genes—*gag, pol, env*—encoding structural and enzymatic proteins have been indicated in light grey. Accessory genes—*vif, vpr, vpu*, and *nef—*are indicated in dark grey. Regulatory genes—*rev* and *tat*—are indicated in black. (**C**) First-generation of HIV-1-based LVs. All HIV-1 proteins, except for Env and Vpu, are included in the packaging plasmid construct (top). The heterologous pseudotyping envelope is provided *in trans* in a separate plasmid (middle). The vector genome encoding the transgene contains intact wt long terminal repeats (LTRs) (bottom). Expression of the packaging and pseudotyping plasmids are achieved via strong constitutive promoters. (**D**) In the second-generation LVs, all accessory proteins are removed, but the system is still *rev* and *tat* dependent. (**E**) In the current third-generation system, *tat* is removed, and essential HIV-1 genes are split into two separate plasmids. Vector genome LTRs are modified to include a 5′ promoter and SIN 3′ U3 element (∆U3). Other *cis-*acting modifications have also been made, including the addition of the Woodchuck hepatitis virus post-transcriptional regulatory element (WPRE), polyA sites, and the central polypurine tract (cPPT). U3: LTR element derived from sequences unique to the 3′ end of the RNA genome; R: LTR element derived from sequences repeated in both LTRs; U5: LTR element derived from sequences unique to the 5′ end of the RNA genome; Ψ: packaging signal; RRE: *rev* response element. Arrows stand for constitutive promoters.

Most retroviruses share similar virion morphology: A nucleocapsid core containing the RNA genome, the capsid surrounding the core containing the three essential enzymes; protease, reverse transcriptase, and integrase, and lastly, the viral envelope that forms spikes protruding from the virion surface [14]. All retroviruses encode three fundamental genes—*gag, pol*, and *env—*which are necessary for the formation of the structural proteins mentioned above [15] (Figure 1A). However, complex retroviruses, including lentiviruses, encode other regulatory and accessory genes. HIV-1, the most studied lentivirus, encodes nine genes: *gag, pol, env, rev, tat, vpr, vpu, vif*, and *nef* [16] (Figure 1B).

### 1.2. HIV-1-Based Lentiviral Vectors

Characterization of HIV-1 not only improved our understanding of the virus but also enabled its adaptation for research and various medical applications, including gene therapy. Gene therapy, first intended for the treatment of inherited diseases, currently has the potential to bring curative interventions to many medical fields. In contrast to traditional protein or chemical-based therapies, gene therapy, in principle, allows for ‘one-shot’ curative benefits following the introduction of correct genetic material into the patients. Therefore, optimal vectors are required for the efficient delivery of genomic materials to targets cells. Owing to reverse genetics techniques, researchers have exploited the ability of viruses to modify mammalian cells and converted them into viral vectors. Lentiviruses are especially popular due to their ability to infect both dividing and slow or non-dividing cells (e.g., stem cells, neurons, muscle cells), their capacity to permanently integrate transgenes into the host cell genome, allowing for long-term stable gene expression, and their low-immunogenic characteristics (compared to that of other vectors, such as the ones derived from adenoviruses) [17].

First, replication-competent retroviral vectors were established based on HIV-1, Rous sarcoma virus, and murine leukemia virus through modifications to the viral long terminal repeats (LTRs) or accessory genes to introduce transgenes of interest [18,19,20]. Further improvements resulted in the development of first-generation replication-incompetent LVs through the splitting of viral components into multiple plasmid constructs [21]. The packaging plasmid contained the structural, enzymatic, regulatory, and accessory viral genes, while the heterologous envelope (usually the glycoprotein of vesicular stomatitis virus, VSV.G) was delivered in a separate pseudotyping plasmid. Several *cis-*acting viral sequences were included in the vector construct to ensure high genome expression during vector production and efficient transduction and integration of vectors (Figure 1C). Long terminal repeats contain identical regions (i.e., U3, R, and U5), which flank the proviral genome, and contain a promoter, enhancer, and polyadenylation motifs, which drive transcription and are vital for reverse transcription [22]. The viral packaging signal, psi (Ψ), is required for packaging of the viral RNA into the virion [23]. In addition, the primer binding site (PBS) and the polypurine tract (PPT) are involved in DNA synthesis during reverse transcription [24].

As researchers started using these initial, or first-generation, LVs for gene therapy applications, the creation of safer vectors became a necessity. Initially, all non-essential accessory genes were removed from the packaging plasmid that was provided *in trans* during viral vector production (Figure 1D). Currently, in third-generation LVs, only genes encoding structural and enzymatic components (i.e., *gag-pol*) and the regulatory *rev* are included (Figure 1E). The LTRs of the viral genome encoding the transgene are substantially modified. While the 5′ U3 element is replaced by a heterologous promoter to drive RNA expression in a Tat-independent manner, most of the transcription factor binding sites are deleted from the 3′ U3 region (i.e., ∆U3), referred to as self- inactivating (SIN) vector design. These SIN vectors, following reverse transcription and integration, minimize the potential of full-length viral RNA production in target cells and, therefore, ensure one round of infection [25]. Several other *cis-*acting modifications to the viral genome have also been made, increasing both the titers and transgene expression levels. The main three elements are the inclusion of the Woodchuck hepatitis virus post-transcriptional regulatory element (WPRE) [26], polyadenylation (polyA) motifs [27], and the central polypurine tract [28].

Since their development, LVs have been in the mainstream of gene and cell therapy applications with notable successes in the clinic (reviewed in [29] and [30]). Recently, Kymriah and Yescarta, two chimeric antigen receptor T (CAR-T) cell therapies, which consist of T cells modified ex vivo via LV interventions, have been approved for use in B cell malignancies in the US [31,32]. Furthermore, currently, there are almost 200 active gene therapy clinical trials using LVs worldwide [33].

## 2. Non-Human Lentiviruses

As mentioned earlier, lentiviruses are a diverse group of viruses isolated from various host species broadly divided into two major groups: primate and non-primate lentiviruses. While there are only two known human lentiviruses—HIV-1 and HIV-2—due to years of major viral cross-species and host-switching transmission [34], there are more than 45 known non-human primate lentivirus (i.e., SIV) subtypes [35]. Notably, HIV-1 and HIV-2 arose as a result of cross-species transmissions to humans from chimpanzees [36] and sooty mangabeys [37], respectively. Known SIV strains demonstrate high species-specific divergence and have been divided into six distinct major subtypes: chimpanzees (SIV_cpz_), sooty mangabeys (SIV_smm_), African green monkeys (SIV_agm_), L’Hoest monkeys (SIV_lho_), Sykes’ monkeys (SIV_syk_), and Colobus guerezas (SIV_col_) [38,39]. Amongst these strains, SIV_smm_ has been dominantly used in LV-based research and, therefore, referred to as the prototype SIV lineage hereafter unless otherwise specified. Although SIV has been demonstrated to be less pathogenic than HIV-1, it displays similar characteristics during infection phases. Interestingly, while SIV infections seem to be benign (i.e., non-pathogenic) in their natural hosts, infection of other simian species results in AIDS-like diseases [40,41]. On the other hand, the non-primate lentiviruses group comprises viruses from several different species, including cats, horses, sheep, cattle, and goats [10,42].

**Figure 2:**
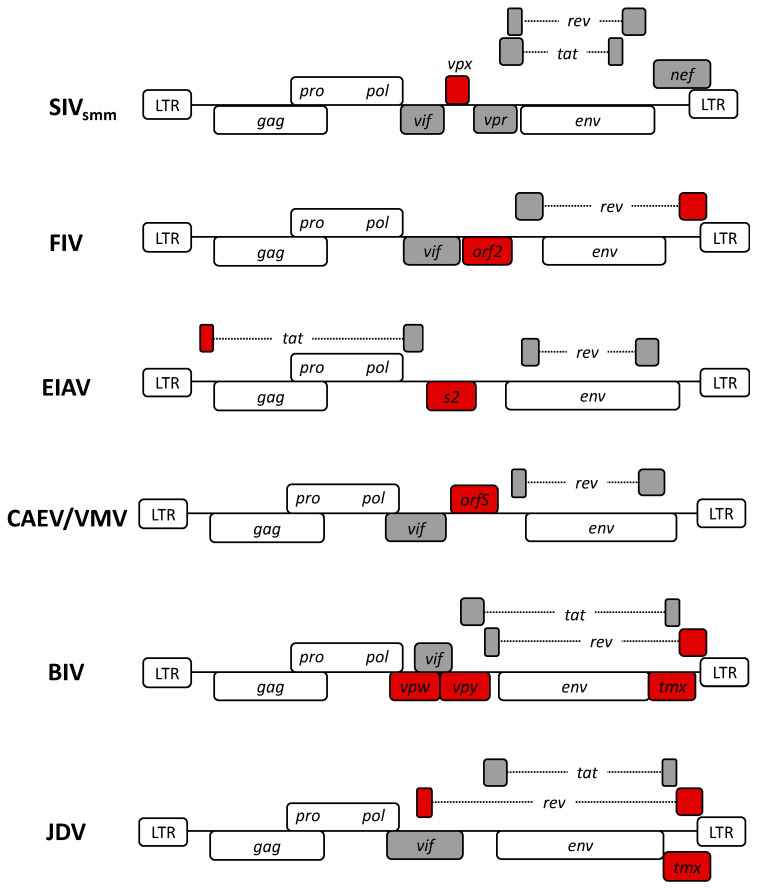
Genomic organization of non-human lentiviruses. Long terminal repeats (LTRs) and essential genes (i.e., *gag, pol, env)* are colored white. Accessory and regulatory genes are colored grey. Notable differences in the genome organization of each lentivirus are indicated in red.

All lentiviruses share similar morphology and genome organization. They have the following *cis-*acting genomic elements: two LTRs, the polypurine tract necessary for reverse transcription, and the packaging signal psi [43,44,45]. In addition, they share functional characteristics, such as the ability to infect terminally differentiated non-dividing cells. Overall, all lentiviruses demonstrate tropism towards cells from the monocyte/macrophage lineages [46,47,48] (an exception to this among non-primate LVs is FIV, which is also able to infect T cells via CD134 (also known as OX40) receptor [49]). However, there are notable differences in the accessory and regulatory proteins encoded as well as their genomic organization amongst different lentiviruses (Figure 2).

Amongst all accessory and regulatory genes, *rev* is the most functionally conserved one, followed by *vif* and *tat.* Typically, *rev* and *tat* genes are encoded by multiple exons towards the 3′ end of the genome. For *rev*, FIV and JDV are notable exceptions. Not only the 3′ *rev* exon in FIV overlaps with the 3′ LTR, but also it contains a unique nuclear export signal compared to other lentiviruses [50]. On the other hand, for JDV *rev*, exons are found farther apart in the genome overlapping *vif* and 3′ LTR, respectively [51]. CAEV and VMV are the only lentiviruses to encode *tat* in a single exon (also referred to as *orfS* [52]), which lacks the transactivator function observed in other lentiviruses [53], while the first exon of EIAV *tat* is located 5′ of the *gag* gene [54]. OrfS is thought to function as a Vpr-like accessory protein, involved in cell cycle arrest during viral infection and replication [55]. In FIV, the transactivator function is conducted by the *orf2* gene [56]. Furthermore, all lentiviral genomes contain *vif* between their *pol* and *env* genes, except for EIAV [57]. *Vif* encodes a protein essential for viral replication and propagation, notably counteracting the effects of host restriction factor APOBEC3G in HIV-1 infections [58]. While EIAV lacks *vif*, it encodes a unique accessory gene: *s2.* While the exact function of the protein encoded by *s2* is still unclear, mutagenesis studies have demonstrated that disrupting its function severely affects viral replication and infectivity of EIAV in inoculated horses [59,60]. It has also been implicated to have an antagonistic function, similar to HIV-1 *nef*, against serine incorporator (SERINC) protein 3 and 5-mediated retroviral restriction in cells [61].

Moreover, *vpr*, an essential accessory gene for HIV-1 bearing crucial roles in the integration of the virus into non-dividing cells and inducing cell-cycle arrest, is conserved only in the primate lentivirus SIV [62]. Yet, its two functions are divided between the SIV *vpr* and a new accessory gene *vpx* in SIV_smm_ (Table 1). In addition, researchers have demonstrated that *vpx* defective SIV_smm_ fails to efficiently infect and replicate in macrophages. Contrarily, *vpr* defective virus could infect macrophages but failed to induce cell cycle arrest [63]. Lastly, there are three other accessory proteins identified only in bovine lentiviruses BIV and JDV (Table 2). *Vpy* and *vpw*, unique to BIV, contain *vif-*like motifs and are thought to have similar functionality [64]. Yet, the function of the *tmx* gene located at the 3′ end of the *env* in both BIV and JDV is currently unknown [65].

Another trademark feature of non-primate lentiviruses is the deoxyuridine triphosphate nucleotidohydrolase (dUTPase) enzyme, which is not encoded by HIV or SIV. While BIV and JDV lack dUTPase, in other non-primate lentiviruses, it is encoded by the *pol* gene [66]. Other retroviruses, amongst other viruses (e.g., herpesviruses [67] and poxviruses [68]), such as murine mammary tumor virus and Mason-Pfizer monkey virus, also have similar functional copies of dUTPase [69,70]. In prokaryotic and eukaryotic organisms, dUTPase plays a crucial role in mitotic cells by driving dUTP hydrolysis. This helps to minimize uracil incorporation into DNA during cell division and hence mutagenesis [71]. It is postulated that the viral dUTPase carries a similar role, preventing the incorporation of uracil into the proviral DNA [72]. As mentioned, while both BIV and JDV lack functional dUTPases, the protein encoded by the corresponding region is still critical for viral replication [73]. Mutagenesis studies have demonstrated that defective dUTPase leads to the incorporation of uracil into the proviral DNA [74] as well as to a delayed replication in non-dividing cells [75,76,77]. Furthermore, infection of animals with defective viruses, in general, leads to reduced viral loads and attenuated disease phenotypes [78,79,80,81].

## 3. Lentiviral Vector Systems Generated Based on Non-Human Lentiviruses

The success of gene therapy interventions ultimately depends on the efficacy and safety of the vectors. Replication-defective vectors, first based on retroviruses and later lentiviruses, have been traditionally preferred over other alternatives to treat several diseases, such as hemophilia. Since the establishment of HIV-1-based LV systems, considerable research has been undertaken to study alternatives from animal lentiviruses. With respect to increased biosafety considerations, the investigation of viruses non-pathogenic for the development of gene transfer vectors has been an attractive option.

### 3.1. Simian Lentiviral Vectors

Simian immunodeficiency virus-based LVs offer certain advantages compared to the other animal lentiviruses. Due to the homology, it shares with both HIV subtypes, SIV has been extensively studied. Initially, replication-competent SIV-based vectors were generated in which the *nef* gene was replaced with the transgene of interest, such as interferon-gamma or green fluorescent protein genes [82,83]. Owing to their attenuated pathogenicity, *nef*-deleted and later *nef*-*vpr* double-deleted SIV vectors were tested as anti-AIDS vaccines, however, with limited success [84,85]. This was rapidly followed by the generation of first and second-generation SIV-based LVs. Similar to the development of HIV-1 vectors, first SIV LVs harbored deletions in the *env* gene or completely lacked it [86,87], and vectors were almost exclusively pseudotyped with the commonly used VSV.G envelope. Several novel approaches involve the design of chimeric vectors using SIV packaging genes and the HIV-1 vector genome [88]. This hybrid system allowed for the generation of Rev-independent vectors as well as the omission of several accessory SIV genes, such as *nef, vpx, vpr*, and *vif*. Cross-packaging of these viruses enabled the production of LVs with comparable titers to that of SIV-only and HIV-1-only systems, while providing an added level of biosafety by decreasing the potential for homologous recombination between genomic sequences from the two lentiviruses [89,90].

Unlike HIV-1, several subtypes of SIV have been utilized and continued to be developed as LV systems. Comparative studies of the systems have revealed that similar to HIV-1, the elimination of accessory genes does not affect vector titers and efficacy [91]. This has led to the development of several third-generation LVs based on two SIV strains: SIV_agm_ and SIV_mac_ (macaques; part of sooty mangabeys clade) [92,93]. While earlier packaging constructs encoded *vif*, *vpr*, *tat*, and *nef*, these new minimal self-inactivation vectors only contain genomic sequences of *gag*, *pol*, and *rev*.

The capacity of SIV-derived LVs to transduce a variety of cell types has been assessed extensively using retroviral and non-retroviral envelopes. Transduction of hematopoietic stem cells [93,94], neurons [95], retinal [96], and lung tissue [97], as well as T cells [87,98] and dendritic cells [99], has been achieved using envelope glycoproteins derived from VSV [87,99], lymphocytic choriomeningitis mammarenavirus (LCMV) [98], murine leukemia virus (MLV), feline endogenous retrovirus RD114 [98], gibbon ape leukemia virus [100], Ebola virus [98], and Sendai virus [92,101]. The choice of envelopes has not only allowed for specific tissue targeting (e.g., lung in the case of Sendai virus) but also conferred advantageous characteristics, such as broad tropism and stability (e.g., VSV.G), and complement resistance (e.g., RD114).

Furthermore, although researchers have sought to remove accessory viral proteins from LV systems due to biosafety concerns, it has been demonstrated that LV tropism and efficacy can be altered by the inclusion of *vpx*. In in vivo studies in mice and rats, SIV-based LVs lacking accessory proteins have demonstrated robust tropism towards neurons [95,102]; however, Hlavaty and colleagues were able to modify the vector to preferentially target glial cells in mice via the introduction of the *vpx* gene in the packaging construct [102]. In addition, the accessory Vpx protein has been exploited to manipulate the early phases of lentiviral infection. Several studies have highlighted that Vpx, encoded by SIV_smm_ and HIV-2, is essential to counteracting innate antiviral restriction factors in myeloid cells, which makes it an absolute necessity when using SIV-based vectors to target myeloid lineages [103,104]. Negri et al. further highlighted that the Vpx-encoding integration-deficient SIV vector could transduce primary human and simian dendritic cells in vitro with greater efficiency [105]. This study, investigating the efficacy of the Vpx-encoding vectors to deliver the reporter green fluorescent protein gene, underlined the promise of integration-deficient LV-based vaccines and clustered regularly interspaced short palindromic repeats(CRISPR)-Cas9 based gene modification strategies. Furthermore, several studies have demonstrated that a similar enhancement of infection can be achieved for heterologous LVs (e.g., HIV-1 and FIV-based) when Vpx protein is provided in trans during LV challenge via non-infectious virus-like particles [106,107].

Simian immunodeficiency virus, similar to human lentiviruses, is subject to innate antiviral restriction. There are several classes of restriction factors identified that target HIV-1 infection in human and simian cells: apolipoprotein B editing complex (APOBEC) family proteins [108], tripartite motif-containing (TRIM) proteins [109], tetherin [110,111], sterile alpha motif- and histidine-aspartate domain-containing protein 1 (SAMHD1) [112], and SERINC [113,114]. As with all primate lentiviruses, SIV has evolved to antagonize these factors. Studies have demonstrated that SIV Vpx and Vpr could degrade SAMHD1 [115], while Nef could antagonize tetherin [116] and SERINC5 [117,118]. Furthermore, it has been demonstrated that only SIV_cpz_ and SIV_smm_ Vif could effectively antagonize human APOBEC3G, highlighting one of the possible reasons why cross-species infection has been only observed with these two strains [119]. Ylinen and colleagues demonstrated that human TRIM5α has minimal activity against a variety of SIV strains [120]; however, recently, a study revealed that TRIM34 could restrict both SIV_agm_ and SIV_smm_ capsids in human monocyte-like cells in a TRIM5α-dependent manner [121]. A similar capsid-specific viral restriction post-entry to the nucleus was observed by Pizzato et al., directed at SIV_smm_ in human blood cells (B and T lymphocytes) [122]. In contrast, human epithelial, primary lung, and CD34+ hematopoietic stem cells have been shown to be permissive for SIV-derived LVs, resulting in robust transduction efficiencies comparable to that of HIV-1 vectors [97,122,123]. In addition, SIV-based vectors have proven invaluable and superior to HIV-1 vectors in the evaluation of SIV and HIV-based hematopoietic stem cell gene therapy strategies in rhesus macaques, owing to their resistance to restriction via rhesus TRIM5α [93,124].

### 3.2. Feline Lentiviral Vectors

Feline immunodeficiency virus-based LVs are the first non-primate vectors to be described [125]. Due to its highly restrictive feline tropism, FIV-based vectors are still considered attractive alternatives to primate LVs (reviewed in [126]). The first-generation of FIV-LVs consisted of a three-plasmid system, where the packaging construct carried a functional *vif* and a non-functional truncated *orf2*.

The initial steps following the development of the vector system addressed questions around pseudotyping, LTR activity in human cells, and the need for wt accessory proteins and cis-acting genome elements. This led to the generation of minimal vector systems devoid of all accessory genes, except *rev* [127,128,129]. Partial *gag* sequences were retained in the vector genome, thought to contain the putative packaging signal [125]. Initially, the production of FIV LVs in human cells was, however, proven difficult due to the lack of promoter/enhancer activity of FIV LTRs. This transcriptional limitation was overcome via the hybrid LTR strategy used for HIV-1-based LVs, replacing the 5′ U3 region with a strong heterologous promoter [127,128].

The VSV.G envelope conferring broad tropism to the LVs has allowed for comparative assessments regarding transduction efficiencies of cells from various species [130,131]. It was established that FIV vectors could transduce many cell types from murine, human, porcine, and feline origins, including neurons [125,132], salivary glands [133], muscle cells [127,134], hepatocytes [134], and dendritic cells [127]. Pseudotyping LVs with less widely used envelopes from arenaviruses (e.g., LCMV) [132], alphaviruses (e.g., Ross river virus) [134], influenza virus (e.g., hemagglutinin from influenza A and GP75 from influenza D) [135], and baculoviruses (e.g., GP64) [136,137,138] has allowed for retargeting of vectors to liver (Ross river virus envelope) and lung cells (influenza, GP64 envelopes), as well as neurons (LCMV envelope) with greater efficacy compared to that of pantropic VSV.G envelope. In addition, FIV vectors have been extensively examined for their suitability for ocular gene therapies. Toxicity and biodistribution studies have been performed in many species, including humans [139,140], monkeys [141,142], mice [143,144], and rabbits [145,146]. Comparative studies have highlighted that FIV LVs could target both the cornea as well as the retina following subretinal and intravitreal injections at levels similar to that of HIV-1-based LVs.

Nonetheless, FIV-based LVs are subject to potent antiviral restriction in human and simian cells, substantially reducing viral vector efficacy and hindering their translational potential. This innate restriction is particularly caused by species-specific variants of tripartite motif-containing 5 α (TRIM5α) protein [147]. Saenz and colleagues demonstrated that rhesus and human TRIM5α, known for its post-entry blocking activity prior to reverse transcription against HIV-1 [109], blocked FIV replication as well [148]. However, for FIV vectors, this could be circumvented by saturating the antiviral activity [149]. Increasing the multiplicity of infection or co-infection with decoy genome-less virus-like particles enables efficient transduction of cells. Although effective in vitro, translation of such strategies to in vivo experiments or the clinic seems impractical and doubtful.

### 3.3. Equine Lentiviral Vectors

Soon after the reporting of LV systems based on FIV, EIAV-based LVs were developed using the split-genome strategy [150]. This first system contained all the accessory proteins of EIAV. However, further improvements were later made to eliminate all accessory proteins, but Rev [151]. Mitrophanous and colleagues also demonstrated that dUTPase could be deleted from Pol without any effect on vector titers [152]. These EIAV vectors could transduce growth-arrested cells and have been successfully pseudotyped with several rhabdovirus [151,153], lyssavirus [152,153], and murine leukemia virus envelopes to varying degrees of efficiency [152].

Using EIAV vectors, Ikeda and colleagues successfully transduced cell lines from several different species [154]. Another study demonstrated that when administered in vivo, EIAV LV could target mouse glial cells and neurons [153]. In addition, in a proof-of-concept study, Yamada and colleagues successfully corrected defects in hematopoietic stem cells from a Fanconi anemia patient using EIAV vectors [155]. Further comparative studies concluded that while EIAV vectors’ transduction efficiencies in several cell lines were comparable to that of HIV-1-based vectors, the transgene expression was unstable and significantly decreased over time [156]. To overcome this, an inducible promoter system was tested [157]. Human and rabbit corneal epithelial cells in the study were targeted more efficiently by EIAV vectors compared to that of HIV-1, and sustained transgene expression was achieved with minimal inflammatory responses.

Extensive studies of the abilities of EIAV vectors led to more developments. Currently, the only non-primate LV packaging cell line is based on EIAV vectors [150,158]. This latest EIAV vector system resembles the third-generation HIV-1-based LVs, owing to the lack of accessory proteins as well as LTR modifications in order to increase transcriptional efficiency and generate self-inactivating vectors. In addition, gag and pol genes are codon-optimized to further minimize sequence overlap and decrease the chance of potential recombination events [158,159]. Using these constructs, a tetracycline (Tet)-regulated packaging cell line has been described where both gag-pol and envelope VSV.G constructs are under the Tet-controlled transcriptional activation (i.e., Tet-on) to reduce protein-specific cytotoxicity and increase viral vector yields. Overall the generated cell line has displayed tight transcriptional regulation and high titers.

The favorable characteristics of EAIV vectors and vector development have advanced EIAV-based LVs into clinical trials for gene therapies targeting the eye and nervous system (ClinicalTrials.gov identifiers NCT01856439, NCT00627588, NCT01505062, NCT01301443, NCT01678872). Of the several clinical trials to date, ProSavin and RetinoStat have been the most successful. RetinoStat is applied for the treatment of neovascular age-related macular degeneration by subretinal administration of dual-transgene encoding EIAV vectors expressing endostatin and angiostatin [160]. A total of 21 patients were enrolled and were administered escalating doses of the viral vector. No adverse effects were noted, and transgene expression was stable up to a year in all patients. Long-term transgene stability was achieved up to 2.5 years in eight patients, while over 4 years in two patients. Although the vector was well tolerated, and the results were encouraging, no meaningful therapeutic correction was achieved in advanced neovascular age-related macular degeneration. ProSavin was developed for patients with Parkinson’s disease [161]. Escalating doses of the vector encoding three dopamine biosynthetic enzymes were administered to 15 patients via bilateral injections into the putamen. Although some adverse effects were reported, no serious events related to ProSavin administration were observed. Long-term follow-up studies demonstrated that the vector was well tolerated in patients for up to 4 years, and statistically significant improvements in motor behavior could be observed in all patients, confirming the therapeutic rationale of the gene therapy application.

### 3.4. Caprine and Ovine Lentiviral Vectors

Gene transfer systems based on goat and sheep lentiviruses have also been described. In the initial attempt of generating CAEV-based vectors, constructs encoding selectable transgenes (e.g., neomycin resistance gene) were generated with intact wt LTRs and stably transfected into goat cells [162]. To rescue vector productions, these cells were then infected with replication-competent CAEV. The titers achieved from this system were significantly lower compared to that of other LV systems. The researchers speculated that this was due to the lack of a packaging signal and a *rev*-responsive element in the vector construct. Mselli-Lakhal et al. showed that the poor vector production was connected to the low expression and accumulation of full-length viral RNA in the cells, leading to defective packaging of the vectors. However, the generation of *env*-defective CAEV vectors pseudotyped with the heterologous VSV.G envelope not only allowed for efficient transduction of human THP-1 and TE671 cells but also led to correct integration and expression of the proviral genome in infected cells [163]. This allowed the production of new viral particles, indicating the suitability of human cells to establish CAEV packaging cell lines. Later, Mselli-Lakhal and colleagues also established replication-defective CAEV vector systems encoding the only gag and truncated versions of *pol* and *env* [164]. Although this system was able to produce functional vectors, viral titers were again unacceptably low (<10^4^ transducing units (TU)/mL).

Meanwhile, Berkowitz et al. reported the derivation of VMV-based vector systems [165]. An initial two-plasmid approach aimed to determine the required genes to establish a human embryonic kidney (HEK) 293 based producer cell line. The incorporation of the hybrid LTR into the viral genome allowed for substantial increases in the viral protein expression. However, neither 293 nor lymphoid CEM cells could be infected using the VMV LVs. Further improvements were made in the vector system, switching to the three-plasmid split-genome strategy. Although RNA expression levels in producer cells were comparable, the transduction efficiency of VMV LVs was approximately 2-logs lower than that of HIV-1 LVs. An investigation into this phenomenon revealed that the low transduction efficiency was associated with viral restriction occurring early in the infection process, namely poor reverse transcription and integration of the viral genome.

### 3.5. Bovine Lentiviral Vectors

Lentiviral vector systems for both JDV and the parental BIV have been described. Metharom and colleagues developed a second-generation replication-defective split genome system based on JDV [166]. This vector had all accessory protein open-reading frames intact. When pseudotyped with heterologous VSV.G envelope, JDV vectors demonstrated approximately 10^6^ TU/mL titers and good transduction profiles in both dividing and growth-arrested HEK 293 and HeLa cells. Alternatively, several groups have derived LV systems based on BIV. Berkowitz et al., the first to report, constructed an earlier generation LV system in which a heterologous promoter-driven transgene was inserted in the *env* gene [167]. When supplemented with VSV.G in trans, the system was able to produce functional vector particles. Several constructs were evaluated for a split genome strategy, allowing for the production of vectors, which could infect a variety of primary cells and cell lines in vitro. Using this vector system, Takahashi et al. could efficiently target murine retinal pigment epithelium cells following a single subretinal injection [168]. They reported robust and stable transgene expression for up to 20 weeks with minimal anti-viral immune responses observed. Improvements to this vector system were made, yielding a third-generation replication-defective LV system, which lacked all BIV accessory genes [169]. Further modifications to the vector genome construct minimized sequence homology with the packaging plasmid, which existed previously [167].

## 4. Conclusions and Future Directions

Lentiviral vectors, with untapped potential, are widely used for gene and cell therapy applications in the laboratories and the clinic. A substantial amount of research has been performed to understand the molecular biology of the lentiviral life cycle due to its advantageous characteristics: relatively large packaging capacity (e.g., compared to that of adeno-associated virus vectors), ability to infect non-dividing cells, allowing for stable transgene expression, and low immunogenicity (e.g., compared to adenoviral vectors). The newer generations of replication-defective LV platforms, lacking accessory genes of wt lentiviruses as well as codon-optimized *gag-pol*, allow for increased safety measures and further minimization of the possibility to generate replication-competent viruses. Although permanent integration of a transgene into the host cell genome is considered a favorable trait, genotoxicity related to viral integration was a significant cause for concern following the adverse outcomes in gammretroviral clinical trials [170,171]. However, owing to the aforementioned safety modifications, current LVs are considerably different than gammaretroviral vectors. In addition, lentiviruses display a distinct integration profile with no preference for enhancer or promoter regions [172,173,174]. Altogether, inherent characteristics of LVs and safety modifications considerably reduce the risk of genotoxicity with no reports of genotoxicity-related adverse effects to date in any of the LV-based gene and cell therapies.

Lack of pathogenicity demonstrated by non-human lentiviruses in humans has made them attractive alternatives for the generation of LV platforms. In addition, the inherent tissue-specific antiviral restriction might provide a biosafety advantage in preventing replication and systemic spread of any potential replication-competent lentiviruses. Shared characteristics amongst lentiviruses and the ability to pseudotype these recombinant vectors with heterologous envelope glycoproteins from other viruses have allowed for targeting of a variety of cell and tissue types. While SIV, FIV, and EIAV have been developed into promising LV systems, VMV/CAEV and BIV/JDV are at their infancy, awaiting several challenges to be overcome. In the case of SIV-, FIV-, and EIAV-derived LVs, based on previous knowledge of and experiences with HIV-1 vectors, packaging systems followed similar iterative modifications: separation of viral genes, removal of accessory and regulatory genes, SIN vector design, and introduction of cis-acting elements, such as WPRE, cPPT, and polyA signals to increase titers and transgene expression.

When utilizing non-human LVs, innate antiviral host restriction responses in human cells is a significant obstacle to be conquered. As previously discussed, factors, such as TRIM5α, APOBEC3G, and SERINC proteins, can substantially curtail the efficacy of LV therapy. SAMHD1 has been implicated in such antiviral activity against HIV-1 and other primate lentiviruses, kinetically restricting lentiviral reverse transcription [175,176,177]. While SIV_smm_ can counteract this activity via its accessory proteins Vpx [178], recently, in a study, Mereby and colleagues demonstrated that non-primate lentiviruses lacked such uniquely gained abilities [177]. Furthermore, in the case of third-generation LVs devoid of all accessory genes, combatting host restriction becomes a crucial concern.

Lentiviral vector’s unprecedented ability to modify the host cell genome has ushered in the era of gene and cell therapies. A deeper understanding of lentiviral biology, as well as the exploitation of reverse genetics, will allow for the generation of a broader portfolio of LV systems to overcome roadblocks and ensure successful translation of advanced therapy medicinal products for clinical applications into the clinic.

## Figures and Tables

**Table 1:** Comparison of accessory and regulatory genes in primate lentiviruses. HIV-1: human immunodeficiency virus type 1; HIV-2: human immunodeficiency virus type 2; SIV_cpz_: simian immunodeficiency virus from chimpanzees; SIV_smm_: simian immunodeficiency virus from sooty mangabeys; SIV_agm_: simian immunodeficiency virus from African green monkeys; SIV_lho_: simian immunodeficiency virus from L’Hoest monkeys; SIV_syk_: simian immunodeficiency virus from Sykes’ monkeys; SIV_col_: simian immunodeficiency virus from Colobus guerezas.

	HIV-1	HIV-2	SIV_cpz_	SIV_smm_	SIV_agm_	SIV_lho_	SIV_syk_	SIV_col_
*rev*	+	+	+	+	+	+	+	+
*vif*	+	+	+	+	+	+	+	+
*tat*	+	+	+	+	+	+	+	+
*vpr*	+	+	+	+	+	+	+	+
*vpx*	−	+	−	+	−	−	−	−
*vpu*	+	−	+	−	−	−	−	−
*nef*	+	+	+	+	+	+	+	+

**Table 2:** Comparison of accessory and regulatory genes in non-human lentiviruses. FIV: feline immunodeficiency virus; EIAV: equine infectious anemia virus; CAEV: caprine arthritis encephalitis virus; VMV: visna-maedi virus; BIV: bovine immunodeficiency virus; JDV: jembrana disease virus.

	SIV_smm_	FIV	EIAV	CAEV/VMV	BIV	JDV
*rev*	+	+	+	+	+	+
*vif*	+	+	−	+	+	+
*tat*	+	−	+	−	+	+
*vpr*	+	−	−	−	−	−
*vpx*	+	−	−	−	−	−
*nef*	+	−	−	−	−	−
*orf2*	−	+	−	−	−	−
*orfS*	−	−	−	+	−	−
*s2*	−	−	+	−	−	−
*vpw*	−	−	-	−	+	−
*vpy*	−	−	-	−	+	−
*tmx*	−	−	-	−	+	+
